# Antigen Binding Characteristics of Immunoglobulin Free Light Chains: Crosslinking by Antigen is Essential to Induce Allergic Inflammation

**DOI:** 10.1371/journal.pone.0040986

**Published:** 2012-07-20

**Authors:** Marco Thio, Tom Groot Kormelink, Marcel J. Fischer, Bart R. Blokhuis, Frans P. Nijkamp, Frank A. Redegeld

**Affiliations:** 1 Division of Pharmacology, Department of Pharmaceutical Sciences, Faculty of Science, Utrecht University, Utrecht, The Netherlands; 2 Division of Medicinal Chemistry, Department of Pharmaceutical Sciences, Faculty of Science, Utrecht University, Utrecht, The Netherlands; Auburn University, United States of America

## Abstract

Beside the production of complete immunoglobulins IgG, IgE, IgA, IgM and IgD, consisting of tetrameric heterodimers of immunoglobulin heavy and light chains, B cells also secrete immunoglobulin free light chains (Ig-fLC). Previous studies showed that Ig-fLCs are able to induce immediate hypersensitivity reactions. It is apparent that recognition and binding of antigen are crucial steps in the onset of these inflammatory responses. In this study, the binding characteristics of Ig-fLC to antigen were further investigated using various biochemical approaches. In addition, we investigated whether antigen-mediated crosslinking of Ig-fLC is required to initiate allergic skin inflammation in vivo. Our study shows that binding of Ig-fLCs to antigen can be measured with different experimental setups. Surface plasmon resonance analysis showed real-time antigen binding characteristics. Specific antigen binding by Ig-fLCs was further detected using immunoblotting and ELISA. Using the ELISA-based assay, a binding affinity of 76.9±3.8 nM was determined for TNP-specific Ig-fLC. Antigen-induced ear swelling in mice passively sensitized with trinitrophenol-specific Ig-fLC was inhibited when multivalent antigen was combined with excess of monovalent antigen during challenge. We conclude that Ig-fLCs are able to interact with antigen, a prerequisite for antigen-specific cellular activation. In analogy to antigen-specific Fc receptor-induced mast cell activation, crosslinking of Ig-fLCs is necessary to initiate a local allergic response.

## Introduction

Immunoglobulins form the backbone of the adaptive humoral immune response. Recognition of a specific antigen can initiate various immune responses directed at removal or neutralization of potential threats. In addition to complete immunoglobulins, free immunoglobulin light chains (Ig-fLCs) are also present in several body fluids and tissues and they have been shown to initiate inflammation by antigen-specific activation of mast cells [1]–[4].

Ig-fLCs contain both a constant and variable region, the latter being defined by gene-rearrangements resulting in antigen specificity. In the tetrameric subunits of Igs, both the heavy chain and light chain variable region contribute to the binding of antigen. Controversy exists on the ability of Ig-fLCs to bind antigen. Diverse studies have found that isolated light chains have no binding capability or the binding strength is several orders of magnitude lower than that of the parent Ig [5]–[7]. On the other hand, other studies showed that Ig-fLCs have the capacity to bind to antigen with reasonable affinities [8]. Also, Masat et al [9] found that the monomeric kappa light chain specific for a molecule expressed by human cells of melanocytic lineage is able to recognize this antigen. Mahana and Schechter [10], [11] proved that preparation of Ig-fLCs by reducing complete antibodies does not influence its antigen recognizing ability compared to natural occurring Ig’s. In several cases, reported affinities are equal to or even exceed those of the parent Ig [12]. The differences in binding affinities of Ig-fLCs might be a consequence of the use of polyclonal Ig fractions to obtain single light- and heavy chains, ineffective renaturation after separation and/or differences in the relative contributions of light and heavy chains in antigen binding by different antibodies.

We have shown in previous studies that antigen-specific Ig-fLCs are crucial in eliciting inflammatory responses leading to contact sensitivity, asthma, IBD, and food allergy in mice [3], [4], [13], [14]. We demonstrated that Ig-fLCs bind and thereby sensitize mast cells. Subsequent contact with the cognate antigen induces mast cell activation and degranulation [14]. This presumes that Ig-fLCs have sufficient binding strength to antigen to trigger receptor activation. In this study, we have investigated the properties of antigen binding by Ig-fLC in more detail using various in vitro binding analysis techniques. Furthermore, we determined if crosslinking of Ig-fLC by antigen is necessary to elicit allergic responses.

## Materials and Methods

### Animal Experiments

All animal experiments were approved by the Animal Ethics Committee of the Utrecht University. Male BALB/c mice (6–8 wks) were obtained from Charles River Laboratories (Maastricht, The Netherlands). The animals were housed in groups not exceeding eight mice per cage. Tap water and chow food were allowed *ad libitum*; there was a 12-h day-night cycle.

### Immunoglobulin Free Light Chains

Trinitrophenol-specific immunoglobulin free light chains were isolated as described earlier [14]. Briefly, trinitrophenol (TNP)-specific IgG1 (kappa isotype) was purified from 1B7-11 (ATCC) culture supernatant by protein G-sepharose (Amersham Biosciences, Roosendaal, the Netherlands). Complete IgG was reduced and alkylated to prevent dimerization. Immunoglobulin free light chains were isolated by gel filtration [14] and stored at −20°C in PBS. Purity of isolated Ig-fLC preparations was checked with SDS-gelelectrophoresis followed by protein staining and/or western blotting. All used Ig-fLC preparations were free of Ig heavy chains (free heavy chain) or intact IgG (data not shown).

### Isolation and Culture of Primary Mast Cells

Primary mouse mast cells were cultured as described earlier [15]–[17]. In brief, femurs from two BALB/c mice were flushed and the bone marrow cells were isolated. Bone marrow cells were cultured for 3 weeks in 10 ng/ml IL-3 and 10% conditioned medium (see below) in RPMI1640 supplemented with 10% fetal calf serum. Bone marrow derived mast cells (BMMCs) were recultured in fresh culture medium every week. As a source of mast cell growth factors, spleen cells were activated with 10 µg/ml pokeweed mitogen (Sigma-Aldrich, Zwijndrecht, the Netherlands). After one week, the cell-free supernatant (conditioned medium) was isolated and stored at −20°C until future use. Bone marrow cultures contain >95% mast cells after >4 weeks of culture [15]–[17].

### Surface Plasmon Resonance (SPR)

Bovine serum albumin (80 µg/ml) was covalently coupled to a Biacore CM5 sensor chip (Biacore AB, Uppsala, Sweden) through the free amino groups by ethyl(dimethylaminopropyl) carbodiimide/N-Hydroxysuccinimide chemistry. Subsequently, one sensor-side was derivatized with 5% picryl sulphonic acid in 0.1 M borate pH 11 for 15 min; the reference side was not treated. The reaction was quenched with ethanolamine. SPR experiments were performed using an Autolab Esprit SPR (Metrohm, Utrecht, the Netherlands) in Hepes-buffered saline pH 7.4 (Hank’s balanced salt (HBS)-buffer) under a constant temperature of 25°C. Regeneration was performed using 10 mM NaOH with 0.1% SDS. TNP-specific free light chains or dinitrophenol(DNP)-specific IgE were added in HBS-buffer at the indicated concentrations and allowed to bind to the surface-bound antigen. Detected binding was corrected for unspecific binding to the sensor chip by subtracting the detected signal measured in the non-treated part of the chip.

### Detection of Antigen-binding by Immunoglobulin Free Light Chains with Immunoblotting

TNP-coupled ovalbumin (TNP-OVA), TNP-coupled bovine serum albumin (TNP-BSA) or unlabeled proteins were electrophoresed on a 12% SDS-polyacrylamide gel and subsequently transferred to a polyvinylidene fluoride (PVDF) blotting membrane overnight. Membranes were blocked with phosphate buffered saline (PBS)/0.1% Tween20/5% non-fat dry milk. Binding to TNP-conjugated proteins was measured by incubating the membranes with TNP-specific Ig-fLC (2 µg/ml) for 2 h at room temperature. Blots were washed with PBS containing 0.1% Tween20 and probed with goat anti-immunoglobulin kappa light chain Ab conjugated to horseradish peroxidase (HRP) (1∶10.000 dilution, Jackson Immunolabs, Suffolk, Great Britain), followed by enhanced chemiluminescence (ECL) detection using ECLplus (GE Healthcare, Zeist, the Netherlands).

### Ig-fLC Binding to Antigen-coated Wells

50 µg/ml TNP-OVA in 0.05 M sodium bicarbonate buffer at pH 9.6 (Sigma Aldrich, Zwijndrecht, the Netherlands) was coated overnight in 96-well ELISA plates (Costar, Sigma-Aldrich, Zwijndrecht, the Netherlands). After incubation and removal of unbound TNP-OVA, unused and charged surfaces in the wells were blocked using PBS/1%BSA for 1 h at 37°C. TNP-specific Ig-fLC was added at indicated concentrations and incubated for 1.5 h at 37°C. After washing with PBS with 0.1% Tween20, binding was detected with a goat anti-Ig kappa light chain antibody conjugated with HRP (1∶10.000) (Southern Biotech, Birmingham, Alabama, USA) in PBS/0.1%BSA. After washing, tetramethylbenzidine (TMB substrate) (Pierce, Rockford IL, USA) was added and incubated at room temperature for color development. Binding curves were analyzed with non-linear fitting analysis (Graphpad Prism 5.04).

### Flow Cytometry

10 mg/ml DNP-HSA (Sigma-Aldrich, Zwijndrecht, the Netherlands) in 0.1 M sodium bicarbonate buffer was coupled to succinimidyl ester activated Alexa-633 (Invitrogen, Breda, the Netherlands) according to the manufacturer’s protocol. To determine binding of antigen to Ig-fLC- or IgE-sensitized mast cells, 2×10^5^ BMMC’s in 200 µl were incubated with 6 µg TNP-specific Ig-fLC or 1 µg TNP-specific IgE for 45 min. at room temperature. Subsequently, 300 ng Alexa633-labeled DNP-HSA was added and incubated for 30 minutes at room temperature. Competition between labeled and unlabeled DNP-HSA was evaluated by adding 10 µg unlabeled DNP-HSA. Binding of DNP-HSA-Alexa633 was analyzed on a FACSCalibur flow cytometer (BD Biosciences, Etten Leur, the Netherlands). To prevent possible degranulation which may interfere with the FACS analysis, all experiments were conducted in 0.05% sodium azide containing buffers to suppress cell activation.

### Ear Swelling Measurements

Right ears of BALB/c mice were intradermally injected with 500 ng TNP-specific Ig-fLCs or DNP-specific IgE in PBS; left ears received PBS only. Twenty hours after injection, 100 µg DNP-HSA or 100 µg DNP-HSA plus 750 µg DNP-L-Ala (Sigma-Aldrich) was injected intravenously in the tail vein. Ear thickness of right and left ears was measured at 10, 30 and 60 minutes after challenge using an electronic engineer’s micrometer (Mitoyo, Veenendaal, the Netherlands). Data are displayed as delta ear thickness for each time point. Ear thickness measured before intravenous antigen injection was used to calculate increase in ear thickness due to antigen challenge. Ear thickness in PBS-injected left ears was measured as control for possible non-specific effects of systemic antigen injection. Each group consisted of 5 animals and the experiment has been performed twice.

## Results

### Antigen Binding of Immunoglobulin Free Light Chains Measured by Immunoblotting

**Figure 1:**
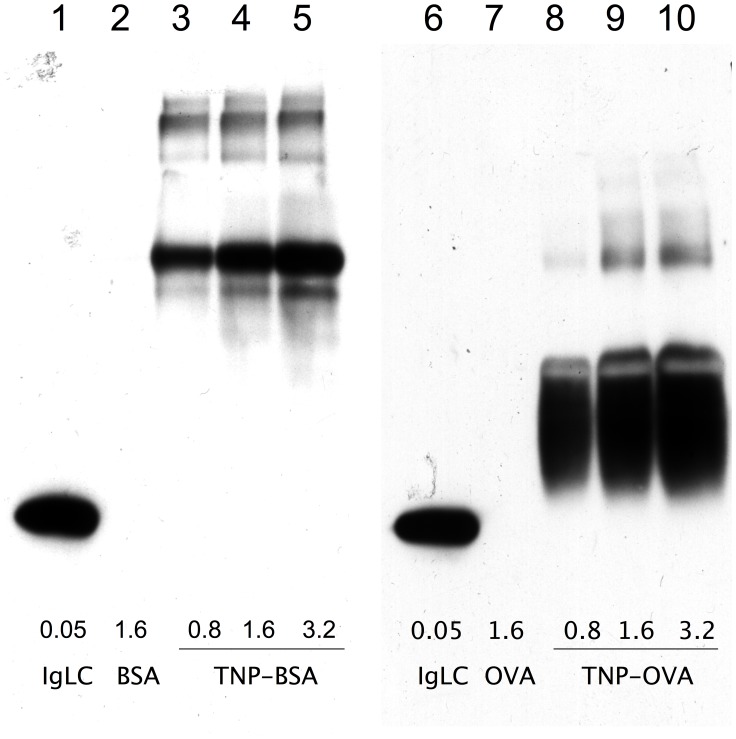
Binding of Trinitrophenol (TNP)-specific immunoglobulin free light chain (Ig-fLC) to TNP-conjugated ovalbumin (TNP-OVA) or TNP-conjugated bovine serum albumin (TNP-BSA) detected by immunoblotting. TNP-coated proteins, TNP-BSA (lane 3–5) or TNP-OVA (lane 8–10), or unconjugated controls (lane 2: BSA and 7:OVA) were subjected to SDS-PAGE and subsequently electroblotted to PVDF membrane. Membrane was incubated with TNP-kappa Ig-fLC (2 µg/ml) and bound Ig-fLC was visualized by HRP-conjugated anti-mouse kappa light chain antibody and enhanced chemiluminescence. Lane 1 and 6 contain Ig-fLC (1 µg) as a positive control for the detection of kappa Ig light chains. Results are representative for three individual experiments.

Binding of Ig-fLC to antigen was evaluated using an immunoblotting technique which has been used earlier to detect immunoreactivity of e.g. IgE [18]. Trinitrophenol-conjugated ovalbumin or bovine serum albumin and corresponding unlabeled proteins were electrophoresed using SDS-PAGE and electroblotted to PVDF membranes. Membranes were probed with TNP-specific Ig-fLC. Binding of TNP-specific Ig-fLC to increasing concentrations of TNP-conjugated proteins was clearly detectable (Figure 1) even after extensive washing. No binding was detected to non-derivatized protein.

### Binding of Ig-fLC to Antigen-coated Microplates

**Figure 2:**
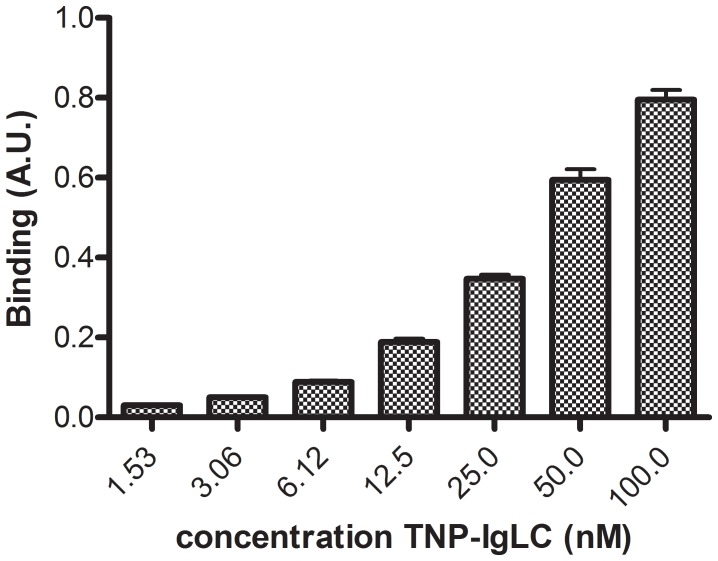
Binding of TNP-specific fLC’s to immobilized TNP-OVA. TNP-OVA was coated to the surface of 96-well plates. Unbound TNP-OVA was washed away followed by blocking with PBS/1%BSA. TNP-specific Ig-fLC was added and binding of Ig-fLC was detected using anti-mouse kappa light chain Ab conjugated with HRP. Results represent 6 individual experiments with duplicate binding curves. Binding curves were analyzed with non-linear fitting (Graphpad Prism 5.04): Kd: 76.9±3.8 nM; R^2^∶0.9905±0.0026; N = 12.

In another experimental setup, binding of TNP-specific Ig-fLCs to antigen was analysed in an ELISA format. TNP-specific Ig-fLC was administrated to TNP-OVA coated 96-well ELISA plates. Figure 2 shows a dose-dependent binding of TNP-Ig-fLC to wells coated with 50 µg/ml TNP-OVA. After non-linear fitting of the binding curves (R^2^∶0.991±0.003, N = 12), the binding affinity of Ig-fLC for TNP-OVA was determined: Kd = 76.9±3.8 nM.

### Surface Plasmon Resonance Analysis of Antigen Binding by Immunoglobulin Free Light Chains

**Figure 3:**
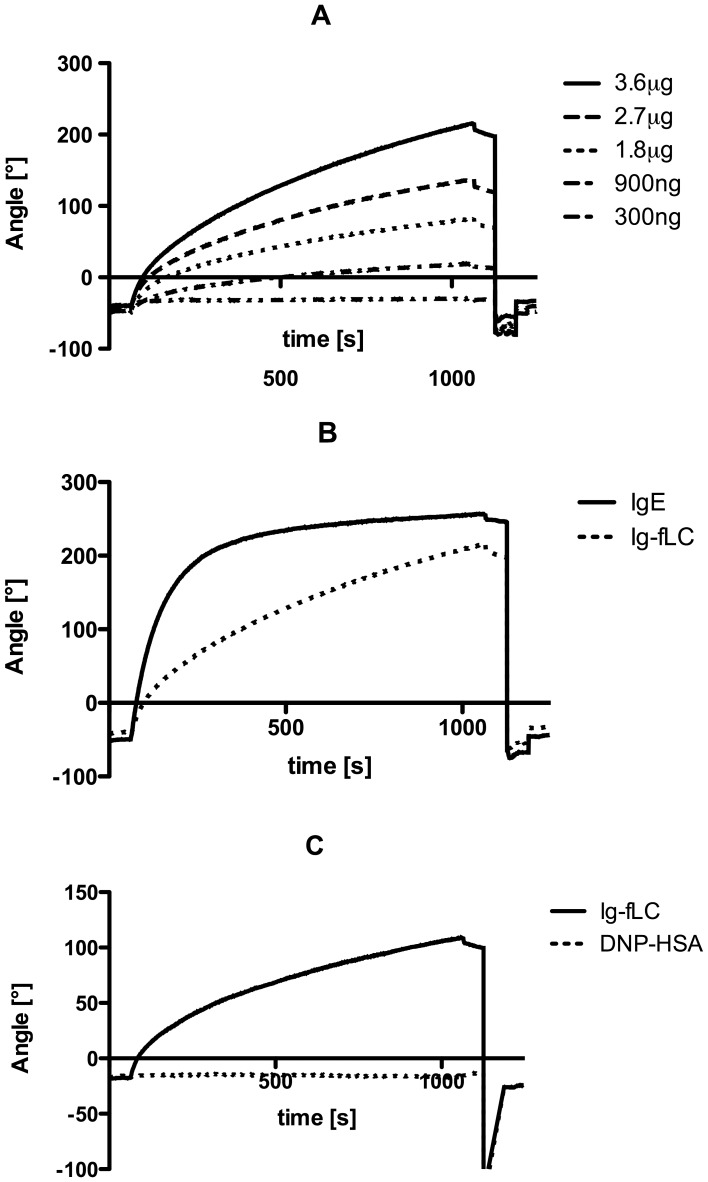
Real-time analysis of TNP-specific Ig-fLC binding to TNP-BSA coated SPR chips. Increasing concentrations of Ig-fLC (300 ng, 900 ng, 1.8 µg, 2.7 µg and 3.6 µg per CM5 sensor chip) resulted in increased binding curves (A). Administration of TNP-IgE (3.6 µg) resulted in significant binding to the TNP-coated surface and saturation around 350 s. Administration of equal amounts of TNP-Ig-fLC showed binding, yet no clear saturation was observed (B). Administration of TNP-BSA (1 µg/ml) completely eliminated the binding of Ig-fLC to the antigen-coated chip (C). No loss in binding was observed after non-conjugated BSA administration (data not shown). Results are representative for 3 individual experiments.

Binding activity of TNP-specific Ig-fLCs for TNP conjugated to albumin (TNP-albumin) was analyzed by surface plasmon resonance (SPR). Figure 3A shows binding curves of various concentrations of TNP-specific Ig-fLC to the TNP-albumin coated surface. Comparable to IgE, Ig-fLC binding shows rapid binding to the antigen. No apparent equilibrium of binding was reached at the indicated concentrations of Ig-fLC even after extending the analysis up to 1500 s (data not shown) (figure 3B). SPR measurements were limited by the available concentrations of TNP-specific Ig-fLC, because high concentrations of Ig-fLC tend to aggregate and results in loss of functional protein. Administration of soluble DNP-conjugated human serum albumin to the reaction chamber completely prevented binding of Ig-fLC to the TNP-coated SPR chip (Figure 3C).

### Immunoglobulin Free Light Chains Bound to Mast Cells are Capable to Recognize Antigen in vitro

**Figure 4:**
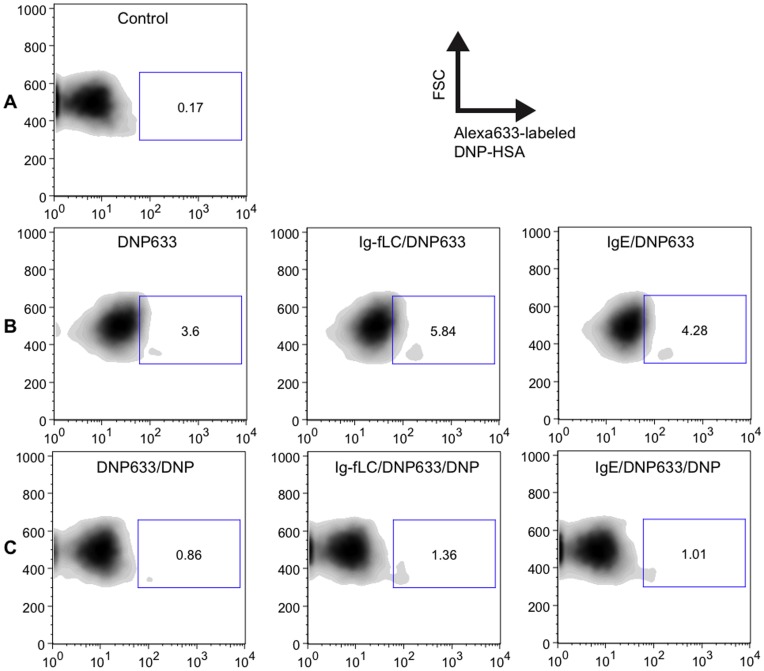
Binding of Alexa633-labeled DNP-HSA to TNP-specific Ig-fLCs bound to mast cells. Murine bone marrow-derived mast cells (BMMC) were incubated with TNP-specific Ig-fLC (6 µg) or IgE (1 µg). After incubation, Alexa633-labeled DNP-HSA (300 ng) was added. Administration of Alexa633-labeled DNP-HSA resulted in increased binding to BMMCs sensitized with TNP-specific Ig-fLC (B, middle) or IgE (B, right) as compared to non-sensitized BMMC (B, left panel). To evaluate whether the binding of A633-DNP-HSA was specific, a 30-fold excess of unlabeled DNP-HSA (10 µg) was administrated simultaneously with the Alexa633 labeled-DNP-HSA. This resulted in a detectable loss in binding to Ig-fLC- and IgE-sensitized BMMCs (C). Panel A depicts untreated BMMC. Results are representative for 3 independent experiments.

To investigate if Ig-fLC bound to the cell surface of mast cells was able to recognize antigen, bone marrow-derived mast cells were sensitized with TNP-specific Ig-fLC. Subsequently, binding of antigen DNP-HSA conjugated with Alexa633 was analyzed using flow cytometry (Figure 4). Incubation of non-sensitized BMMC with A633-labeled DNP-HSA (Fig. 4B, left panel) resulted in some background staining compared to non-treated BMMC (Fig. 4A). BMMCs sensitized with TNP-specific Ig-fLC (2.4E-10 mole) and IgE (4E-12 mole) (Fig. 4B, middle and right panel) showed an increase in binding vs. non-sensitized BMMCs (Fig. 4B, left panel). To evaluate whether the binding of A633-labeled DNP-HSA to BMMCs was specific, binding of A633-labeled DNP-HSA was measured in presence of a 30-fold excess of unlabeled DNP-HSA. This resulted in a reduction in bound A633-labeled DNP-HSA to Ig-fLC- or IgE-sensitized BMMCs (4C, middle and right panel).

### Crosslinking of Ig-fLC is Necessary to Induce Skin Inflammation in vivo

**Figure 5:**
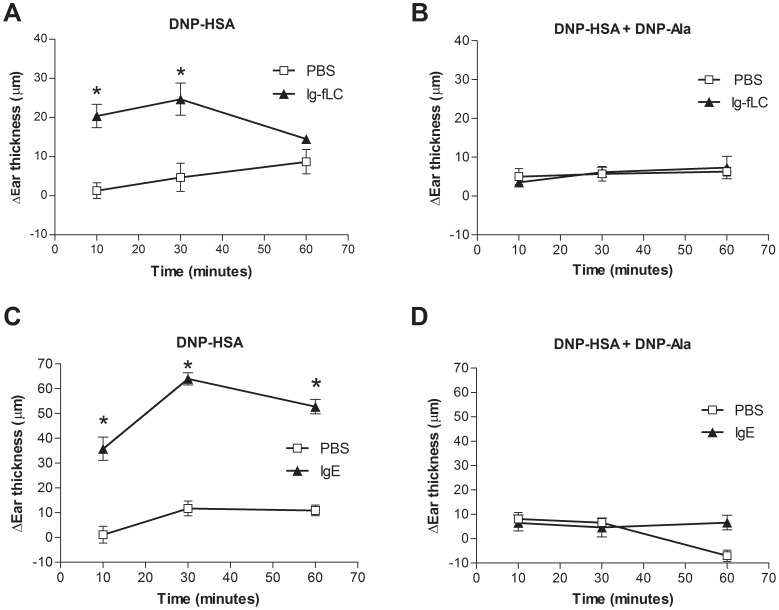
Crosslinking is required for elicitation of allergic ear swelling responses. Mice were intradermal injected in the right ears with either 500 ng TNP-Ig-fLC (A and B) or 500 ng TNP-specific IgE (C and D). At 20 hours after sensitization, mice were intravenously injected with either 100 µg DNP-HSA (panel A and C) or 100 µg DNP-HSA combined with 750 µg DNP-Ala (50× molar excess monovalent antigen) (panel B and D). Ear swelling was measured at 10, 30 and 60 minutes after antigen injection and compared to ear thickness before antigen challenge. DNP-HSA administration caused a significant increase in ear swelling in both Ig-fLC and IgE sensitized mice (panel A and C). Challenge with DNP-HSA combined with DNP-L-Ala prevented development of ear swelling for both Ig-fLC- (panel A vs B) and IgE-mediated (panel C vs D) responses. All treatment groups consisted of 5 mice. Values are mean±SE.

It is well-described that IgE-mediated activation of mast cells requires crosslinking of IgE bound to the high affinity IgE receptor FcεRI [19]–[21]. To investigate if a similar crosslinking of Ig-fLC by antigen is required to induce a local allergic response, right ears of mice were intradermally sensitized with TNP-specific Ig-fLC or DNP-specific IgE as a positive control. Left ears were injected with PBS. Systemic challenge with multivalent antigen: DNP-conjugated human serum albumin (DNP-HSA: 30–40 molecules DNP per molecule albumin) resulted in a rapid ear swelling of Ig-fLC sensitized (Fig. 5, panel A) and IgE-sensitized ears (Fig. 5, panel C). No ear swelling was induced in PBS injected left ears. When mice were challenged with DNP-conjugated albumin in combination with a 50× excess of monovalent antigen (DNP-L-Ala), no ear swelling was observed in Ig-fLC-sensitized mice (Fig. 5, panel B). As expected, presence of excess of monovalent antigen also prevented the antigen-induced IgE-mediated ear swelling response (Fig. 5, panel D). When mice were challenged with DNP- L-Ala only, also no induction of ear swelling was observed (data not shown).

## Discussion

In this study, we show using various binding assays that Ig-fLCs bind specifically to cognate antigen. Furthermore, flow cytometric analysis shows that Ig-fLCs, also when bound to the mast cells, are able to bind antigen. Next, it is shown that crosslinking of Ig-fLC by multivalent antigen is required to induce an allergic skin inflammation. These results suggest that Ig-fLC-induced allergic responses bear similarities with these elicited through crosslinking of the FcεRI-bound IgE by allergens.

Three different experimental setups were used to investigate the interaction of antigen-specific Ig-fLC with its cognate antigen. First, the interaction of hapten-specific Ig-fLC with hapten-conjugated proteins was measured by immunodetection of binding to membrane immobilized antigen. This method has been proven useful to characterize binding of IgE to specific allergen epitopes [18], [22], [23]. Binding of specific Ig-fLCs to Ag-conjugated and not to non-derivatized protein was clearly detectable using this approach, which indicates that binding affinity is sufficient to survive repeated washing. Secondly, concentration-dependent binding of Ig-fLC to antigen-coated wells was measured in an ELISA format. Thirdly, real-time binding of immunoglobulin free light chains to antigen was measured with SPR. Ig-fLCs showed rapid binding to antigen. However, binding curves did not reach equilibrium which made calculation of associating and dissociating constants not possible. The continuous increase in binding signal suggests that after the initial binding to the antigen, a continued non-typical binding and/or aggregation of Ig-fLCs to the chip surface occurred. Notably, such non-saturating binding kinetics were also reported for binding of C-reactive protein to FcαRI [24]. Taken together, the different binding assays consistently support earlier observations that Ig-fLCs bind specifically and with sufficient strength to antigen [7], [25]. Although affinities may not reach those of tetrameric antibodies, antigen-bound Ig-fLC resisted extensive washing indicating a tight binding, perhaps due to increased avidity or conformational changes upon binding. In this study, the K_d_ for binding for TNP-specific Ig-fLC was found 76.9±3.8 nM, demonstrating a moderate-to-high affinity for the antigen. A wide range of binding affinities ranging from 10^−3^ till 10^−8^ M have been reported, and even some studies showed that Ig-fLCs are not capable to recognize and bind antigens [26]. The reported discrepancies may be due to the way the Ig-fLCs are prepared, assays used to determine binding and the antigen involved [11], [27]–[29]. Thus far, limited studies on the measurement of specific Ig-fLC in body fluids have been reported. Nakano et al. were unsuccessful in the measurement of specific free Ig light chains as a diagnostic marker for patients with allergy, although practical limitations may have hampered this study [5], [30]. On the other hand, in AIDS patients with Toxoplasma gondii encephalitis, Toxoplasma gondii encephalitis-specific free kappa light chains were successfully detected in cerebrospinal fluid [31]. Whether the currently employed methods are useful to measure antigen-specific Ig-fLC in body fluids and/or identify to what antigens Ig-fLC are directed is subject of ongoing studies.

In previous studies, fluorescence microscopy and rosetting assays showed that Ig-fLC bind to primary cultured mast cells [14]. Furthermore, studies in mast cell-deficient mice showed that Ig-fLC-induced allergic responses were mast cell-dependent. To stimulate an antigen-specific activation of mast cells, it is required that membrane (receptor) bound Ig-fLC is able to bind to antigen. We used flow cytometry to analyze antigen-binding by cell membrane-bound Ig-fLC and demonstrated that primary cultured murine mast cells sensitized with TNP-specific Ig-fLC bind antigen. Such recognition and binding of antigen allows Ig-fLC to elicit activation of mast cells in an antigen-specific manner similar to intact immunoglobulins such as IgE.

It is well-described that antigen-specific mast cell activation via the FcεRI or FcγR receptors requires crosslinking of multiple receptor-bound immunoglobulins [19]–[21]. In case of sensitization with monoclonal Ig (e.g. hapten-specific IgE) crosslinking can be accomplished via multivalent antigen. Although in previous experiments an involvement of receptors associated with the common gamma chain such as Fc receptors has been excluded [14], it was thus far not known whether Ig-fLC elicited allergic responses showed analogy with IgE in the requirement of crosslinking by antigen to provoke mast cell activation. In present study, it is shown that crosslinking of Ig-fLCs -by multivalent antigen- is necessary to induce a skin allergic reaction in a passive cutaneous anaphylaxis experiment. Moreover, when multivalent antigen is combined with an excess of monovalent Ag, no Ig-fLC-induced ear swelling is found. As expected, similar responses are found for IgE-induced allergic skin responses. This suggests that the mechanism of Ig-fLC-mediated mast cell activation and initiation of local inflammatory responses shows similarities with activation induced by Fc-receptors [32].

In conclusion, in this study we have further explored the interaction of a hapten-specific Ig-fLC with its cognate antigen. It is shown that binding of Ig-fLC is of sufficient strength to be detected with common immunodetection methods. Once bound to mast cells, Ig-fLCs are able to interact with antigen, a prerequisite for antigen-specific receptor activation. In analogy to antigen-specific Fc receptor-induced mast cell activation, crosslinking of Ig-fLCs is necessary to initiate a local allergic response.
